# Impact of Pandemic on European Well-Being: Visualizing Scenarios from the SHARE Database

**DOI:** 10.3390/ijerph18094620

**Published:** 2021-04-27

**Authors:** Aurea Grané, Irene Albarrán, David E. Merchán

**Affiliations:** Statistics Department, Universidad Carlos III de Madrid, 28903 Getafe, Spain; irene.albarran@uc3m.es (I.A.); merchanc.david@gmail.com (D.E.M.)

**Keywords:** clustering analysis, *k*-prototypes, mixed data, pandemic, SHARE survey, vulnerability profiles, well-being

## Abstract

The objective of this study is to analyse the effect of a pandemic shock on the well-being of the European population aged 50 or over. Data comes from wave 7 of the Survey of Health, Ageing and Retirement in Europe (SHARE), carried out in 28 countries and representing over 170 million aged individuals in Europe. We start by designing two indicators in order to capture the risk of being unhealthy and economically vulnerable; next, we combine them with socio-demographic information and obtain the vulnerability profiles by means of the *k*-prototypes clustering algorithm. Subsequently, we design a shock similar to the COVID-19 pandemic and measure its effects on the vulnerability profiles. The results suggest that the average level of economic and health vulnerability is relatively low, although levels differ across European regions, with the most vulnerable being Eastern European countries. It was observed that the shock most affected countries with a greater proportion of individuals initially deemed vulnerable in terms of mental and physical health, as well as countries where tourism and retail sectors are the most vital for their economies. These findings led us to conclude that public policies should be differentiated by European regions, and Governments must establish action plans in order to better meet the physical and mental health needs of their citizens, as well as addressing monetary poverty and financial difficulties.

## 1. Introduction

According to Eurostat [1] the percentage of population in the European Union older than 50 years of age is 40.9%. The World Health Organization (WHO) estimates that Europe will have one of the oldest populations in the world by 2050, with 30% of the population over 60 years old; additionally, life expectancy will be close to 80 years [2]. This means that older people will constitute a proportionally large population and will demand inclusion, as well as socioeconomic and health care. For these reasons, European institutions are designing policies that include the older population. For example, in the framework of the UN’s 2030 Agenda for Sustainable Development, Sustainable Development Goals (SDGs) were laid out, some of which were related to the older population. For instance, Goal 1 “End poverty in all its forms everywhere” and Goal 8 “Decent work and economic growth” support the older population with policies related to retirement, tax-funded minimum pension, education for older workers, social assistance; Goal 2 “End hunger achieve food security and improved nutrition, and promote sustainable agriculture” aims to improve nutrition, i.e., through a diet with vitamins, minerals and proteins; Goal 3 “Ensure healthy lives and promote well-being for all at all age” aims to guarantee a health system that cares about complex and chronic disease using a prevention, promotion, treatment and rehabilitation approach. Other goals like quality education (Goal 4), gender equality (Goal 5), and sustainable cities and communities (Goal 11) aim to reduce the vulnerability of this group.

At present, we are suffering a pandemic due to SARS-CoV-2 (COVID-19). Although this virus can affect any age group, it affects older people with greater intensity, especially those who have pre-existing medical diseases such as lung or heart disease, diabetes or cancer [3]. Therefore, the risk for severe illness with COVID-19 increases with age, with older adults at highest risk (The Center for Disease Control and Prevention (CDC), 2021. https://www.cdc.gov/coronavirus/2019-ncov/need-extra-precautions/older-adults.html (accessed on 25 March 2021)).

The Center for Disease Control and Prevention (CDC) [4] mentioned that in USA “people in their 50s are at higher risk for severe illness than people in their 40s. Similarly, people in their 60s and 70s are, in general, at higher risk for severe illness than people in their 50s. The greatest risk for severe illness from COVID-19 is among those aged 85 or older.” In March 2021, the CDC posted a graph where it was observed that in USA, there were a total of 1117.3 hospitalisations per 100,000 individuals over 50 years of age.

Health authorities all over the world are warning older people that they are at a higher risk of more serious and possibly fatal symptoms associated with COVID-19. Mortality data from the Oxford COVID-19 Evidence Service indicated a risk of mortality of 3.6% for people in their 60s, which increases to 8.0% and 14.8% for people in their 70s and 80s (Oxford COVID-19 Evidence Service, University of Oxford, https://www.cebm.net/oxford-covid-19-evidence-service/ (accessed on 25 March 2021)).

The severity and outcome of COVID-19 largely depends on a patient’s age. Adults over 65 years of age represent 80% of hospitalisations and have a 23-fold greater risk of death than those under 65 [5]. COVID-19 has so far killed more than 2,750,000 people (2,758,733 by the time this paper was written), with the majority of deaths (73%) occurring in people over the age of 65 (Worldometer, 2020. https://www.worldometers.info/coronavirus/coronavirus-age-sex-demographics/ (accessed on 25 March 2021)) (See Ref. [6])

COVID 19 has caused a marked increase in deaths worldwide, but with significant variation between countries and causes of death. The total amount of excess mortality will also depend on the age structure of a population. Countries with age structures weighted towards an older population will experience higher mortality those with an age structure weighted towards a younger population.

This pandemic has challenged healthcare systems around the world in a way that has not been seen in modern times. Older people are the most affected by the pandemic as a group with the highest risk of hospitalisation and death (Williamson E, Walker AJ, Bhaskaran KJ et al. (2020) OpenSAFELY: factors associated with COVID-19-related hospital death in the linked electronic health records of 17 million adult NHS patients. https://www.medrxiv.org/content/10.1101/2020.05.06.20092999v1 (accessed on 25 March 2021)) [7,8]. Research is a central component of the global response to COVID-19 [9], as the pandemic has had a huge impact on our ability to design and conduct research relevant to older people, not just COVID-19. So, one of the biggest challenges for senior research is currently COVID-19.

This pandemic is disrupting lives and communities in the United States and around the world [10,11]. As the impacts of the disease continue, the economic consequences of the pandemic and the responses to it begin to manifest themselves, as jobs are lost, businesses close and financial markets falter. While the implications of the current economic downturn for older adults are only just beginning to emerge, past experience suggests that the consequences could be devastating for many [12].

Elderly people and those with pre-existing chronic health conditions may be at higher risk of developing severe health consequences from COVID-19. In Europe, this is of particular relevance to aging populations living with noncommunicable diseases, multimorbidity and frailty [13].

This pandemic constitutes an extraordinary global health, social and economic challenge. The impact on people’s mental health and the psychological well-being of vulnerable groups (health workers, outpatients or the elderly among others) is expected to be high [14]. People who have or had symptoms related to COVID-19 or various disadvantaged socio-economic backgrounds are at significantly higher risk of general psychiatric disorders and loneliness. It is important to consider the broader psychological impact of the pandemic on a wider population. During the first wave, almost a third of the population was affected by COVID-19 in various ways and to different degrees [15]. The COVID-19 outbreak has evolved into a global public health crisis with an increasing numbers of people affected psychologically in various ways and to varying degrees (see [15,16,17] among others).

This pandemic, as well as the public health and emergency measures implemented to reduce contagion, have profoundly changed people’s lifestyles and are believed to be a threat to physical and mental wellbeing. Factors such as the long duration of quarantine, fears of infection, inadequate information, financial, and job loss have been associated with a greater negative psychological impact [14,18,19]. According to [18], depression is a normal reaction to a sudden worsening in living conditions, involving isolation and uncertainty about economic expectations and life expectations in general.

With these facts in mind, we can infer that older people are vulnerable in economic, social, educational, gender, and health terms, but they may be more affected by health issues like pandemics. Therefore, it is important to generate knowledge about the condition of vulnerability of the elderly. For that reason, the main objective of our research is to analyse the impact of a pandemic on the well-being of European people older than 50 years of age. For this purpose, we designed two indicators that can capture the risk of being unhealthy and economically vulnerable and combine them with socio-demographic information in order to obtain vulnerability profiles.

The paper proceeds as follows. In Section 2 we describe the data used for this study, the methodology to construct the indicators and the process to obtain the vulnerability profiles. Section 2 also contains the design of the pandemic shock. In Section 3, we analyse the results by visualising the indicators, the vulnerability profiles and the pandemic shock. Finally, we conclude in Section 4.

## 2. Data and Methods

In this section, we describe the construction of two indicators which are useful to measure the economical and health vulnerability of the elderly. They are based on information available in the SHARE dataset. Next, we present the process to obtain the vulnerability profiles, and finally, we explain the design of the pandemic shock to be applied to the SHARE dataset, with the aim of studying its effect on the vulnerability profiles and detect possible deficiencies on national social protection systems.

### 2.1. Descriptive Analysis and Design of Indicators

#### 2.1.1. Data

In this work, we use data from the Survey of Health, Ageing and Retirement in Europe (SHARE), which is a cross-national and multidisciplinary panel database of microdata related to topics like health, socio-economic status and social and family networks of about 170 million individuals aged 50 or older. See also http://www.share-project.org/home0.html for more details (accessed on 1 April 2020) on this project.

In particular, we use SHARE survey—Wave 7. This survey was implemented using the web in 2017 in Austria, Germany, Sweden, Netherlands, Spain, Italy, France, Denmark, Greece, Switzerland, Belgium, Israel, Czech Republic, Poland, Luxembourg, Hungary, Portugal, Slovenia, Estonia, Croatia, Lithuania, Bulgaria, Cyprus, Finland, Latvia, Malta, Romania, and Slovakia. This last wave contains questions about activity, physical health, retrospective health care, retrospective accommodation, behavioural risks, retrospective children, retrospective employment, cognitive functions, children, employment, and pensions, financial transfer, health care, household income, housing, mental health, and social support. In this work, we use data from wave 7 to achieve the proposed objective.

The target of this survey are all persons aged 50 or more, with regular domicile in one of the aforementioned countries. People who are incarcerated, hospitalised or out of the country, unable to speak or have moved to an unknown address were excluded from the sample [20]. Since it is a longitudinal survey, all the respondents interviewed in previous waves were part of the sample of wave 7. The survey has calibrated cross-sectional weights, which were computed by country to match the size of national target populations. For each country, a logit specification of the calibration function and calibration margins that consider gender and age groups were used. This information for the calibration was taken from the EUROSTAT regional database.

For our research we only considered European countries, so we removed Israel from the database and kept only those individuals that had a calibration value. After that, the dataset of study consists of 73,274 complete cases that represent 171,585,757 European citizens.

Regarding survey information, we use 30 variables recoded in the modules of Demographics, Physical Health, Mental Health, Health Care, Employment and Pensions, Housing, Household Income, Consumption, Activities and Expectations.

We split the variables into two groups, that we call descriptive variables and thematic blocks. The first group consists of sociodemographic information and contains variables such as country, gender, age group, marital status, and education. These variables do not contribute to the indicators, but will be used to create the vulnerability profiles. In this paper, we consider two thematic blocks: one related to economics and the other to health. The variables included in these blocks contribute to the indicators and are described below.

Due to the sample design of this survey and the weight of each individual, we used the R-package survey by Lumley [21] to perform the statistical analysis.

#### 2.1.2. Indicators

Our first aim is to measure the condition of vulnerability of the elderly by means of two indicators: the first is related to economic situation and the second to health condition. To build them, we use the information available on SHARE dataset and follow the protocol described in Grané et al. [22], designed to create indicators from mixed data. In particular, we first identify those variables in the dataset that measure relevant aspects to be studied and group them in thematic blocks. Next, in each thematic block, variables are grouped in subthemes, binarised following the recommendations of related literature, always respecting the same polarisation, and each particular subindicator is obtained by aggregation. Finally, indicators are created by adding the corresponding subindicators and rescaled to 0–10; value 0 means no vulnerability at all and value 10 indicates maximum level of vulnerability. Note that all variables have the same contribution to each particular indicator. Thus, grouping variables in subthemes is optional, although it may help to better interpretation.

This design has several advantages: First, we can use both numerical and categorical variables, which can be measured on different scales; second, the binarisaton of variables is useful to unify scales and to identify different patterns; finally, indicators are additively constructed, so that relevant new variables can be added if necessary.

In what follows, we give a detailed description of the construction of the indicators.

##### Economic Indicator

This indicator is compounded by four subindicators: Monetary poverty, Unemployment, Economical Distress, Weekly consumption.

Monetary poverty subindicator measures whether the individual household is in a situation of monetary poverty. It takes value 1 if the individual income is less than the 60% of the national median income after social transfers, otherwise takes the value 0. The threshold is computed by Eurostat for each European country and we took this value for the year 2018. The individual income is computed with the variable *thinc2*, which calculates the total household net income, and we normalise it by considering the household composition according to OECD modified scale [23] (This assigns a value of 1 to the household head, of 0.5 to each additional adult member and of 0.3 to each child under 14 years old).

The Unemployment subindicator measures whether the individual is unemployed. It takes value 1 when the individual is unemployed and 0 otherwise. We use variable cjs that asks for the current job situation. We compute this subindicator because these people do not receive income, and this could cause them to be unable to buy food, save or pay for their health or retirement.

The Financial Distress subindicator lets us know whether the individual has enough income to be able to make ends meet. To create this subindicator, we used the variable fdistress that is computed from the question “*thinking of your household’s total monthly income, would you say that your household is able to make ends meet*”, the possible outcomes are 1-With great difficulty, 2-With some difficulty, 3-Fairly easily and 4-Easily. In this case, the subindicator takes value 1 when the answer is 1-With great difficulty or 2-With some difficulty, and it takes value 0 otherwise.

Finally, the Weekly consumption subindicator measures vulnerability in terms of how often the individual consumes legumes, beans, eggs, meat, fish or poultry. To create this subindicator, we use two variables: the first one, *legeggs,* measures how often the individual consumes legumes, beans or eggs, while the second one, *meat,* measures how often the individual consumes meat, fish or poultry. For both variables, the possible outcomes are Not applicable, Less than once a week, Once a week, Twice a week, 3–6 times a week, and Every day. We assign a numerical value for each possible outcome (from 0 to 4) and add both variables obtaining a new one that takes values between 0 and 8. Using this information we define a threshold in which if the value is less than or equal to 2, we consider the individual as vulnerable, which means that in the weekly consumption subindicator the outcome of this individual is 1 or less.

**Figure 1:**
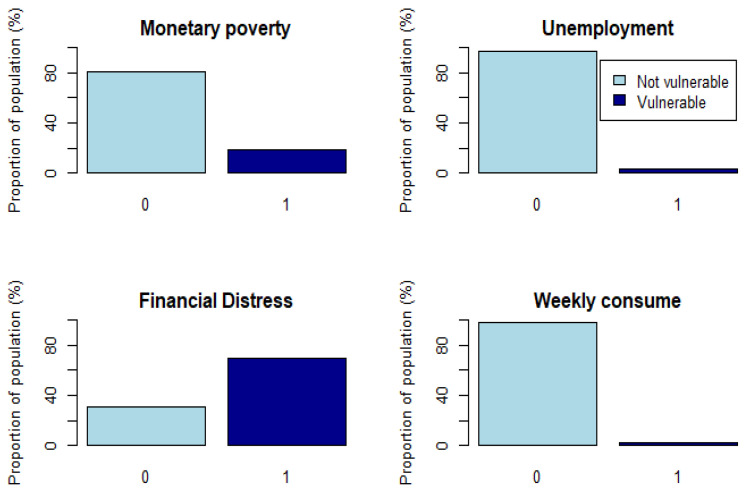
Economic subindicators distributions.

In Figure 1, we can see a bar plot for each subindicator with their distributions. The only subindicator where the group of vulnerable people is greater than the nonvulnerable is that of financial distress. This may happen because it is a self-reported question, also Pettinicchi & Börsch-Supan [24] mention using SHARE data that formerly self-employed retirees have lower incomes and they report high levels of financial distress.

**Figure 2:**
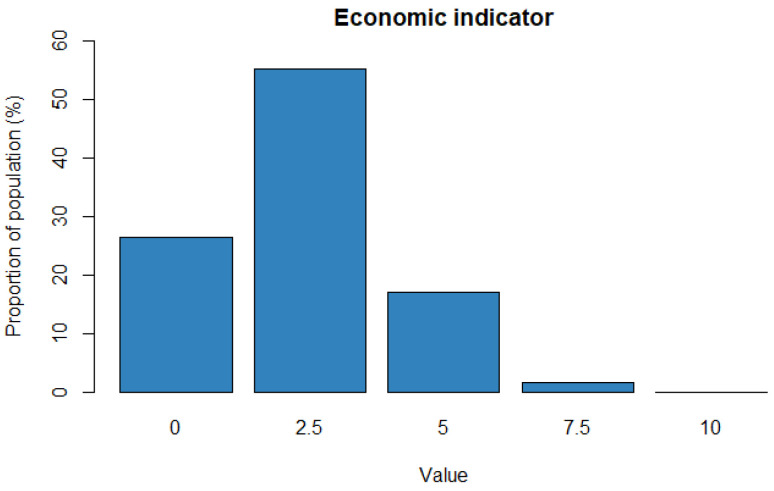
Economic indicator distribution.

The economic indicator is obtained by adding the previous subindicators and rescaling to 0–10. The indicator mean value is 2.33, the standard deviation is 0.01 and the median is equal to 2.5. This means that, in general, Europe has a low level of economic vulnerability, since there are low observations with the maximum levels of vulnerability. This makes sense because Europe is recognised as a continent with a strong economy with a market of 27 countries that make up the European Union. Figure 2 shows the distribution of the economic indicator.

##### Health Indicator

This indicator evaluates the vulnerability in terms of four subindicators: General health, Mental health, Physical health, and Health care.

The General health subindicator is compounded by two variables. The first one is *sphus* which measures Self-perceived health using de US scale; this scale includes Excellent, Good, Fair, Poor, Very Poor. In order to design the subindicator, we consider as vulnerable those individuals that answer Fair, Poor or Very Poor, which means the subindicator takes value 1 whenever those responses take place. The second variable used is *chronic*, which is a numerical variable that counts the number of chronic diseases; in this case we used as a threshold 2 chronic diseases following the research of Costa-Font et al. [25]. Thus, if the interviewee has 2 or more chronic diseases, they are considered as a vulnerable (it takes the value 1 in the binary transformation). We added these two binary variables to compute the General health subindicator.

The Mental health subindicator measures the vulnerability in terms of three variables. First, eurod uses the EURO depression scale 0–12 to measure whether the individual feels depressed or not. We binarise this numerical variable following the work of Costa-Font et al. [25], in which they classified a respondent with a 4 or less on the EURO depression scale as a 0 (not depressed/not vulnerable), else a 1 (depressed/vulnerable). The second variable used is lifesat, which is a numerical variable that uses a scale between 0 and 10, where 0 is completely dissatisfied and 10 completely satisfied; in this case, we classify as vulnerable those people that answer in the scale a value less than 7. The last variable used was lifehap, a categorical variable that measures the life happiness through the question: “*How often, on balance, do you look back on your life with a sense of happiness?*” in which the possible outcomes are Often, Sometimes, Rarely, Never. In this case, we classified a person as vulnerable if his/her responses were Rarely or Never, which means that the binarised variable takes value 1 when these responses occur. For all the above, the subindicator is the sum of the three binarised variables.

The Physical health subindicator considers five variables related to mobility limitations, limitations with activities of daily living and the body mass index (BMI). Mobility limitations issues are measured using two variables Gali and Mobility, the former classifies people as being limited or not limited in mobility, while the latter measures how many difficulties the person has within a range between 0 and 10. We consider a person as vulnerable if he or she has at least one mobility limitation. Likewise, we used adl and iadl variables, which are used to measure the functionality in terms of personal and instrumental activities of daily life. Both variables are numeric, the first one with a range between 0 and 6, while the second one takes values between 0 and 9. Like the previous variables, if the person has at least one limitation is classified as vulnerable. The last variable used for this particular subindicator is the BMI, a very popular index whose categories are established by the following ranges: less than 18.5 (underweight), from 18.5 to 24.9 (normal weight), from 25 to 29.9 (overweight) and greater than 30 (obesity). We binarised the BMI defining as vulnerable those individuals who are underweight, overweight or obese. For all the above, the subindicator is the sum of the five binarised variables.

Finally, the Health care subindicator measures how the elderly population has been taken care of and has used the health system. To create this subindicator, we use the variable doctor that measures how many times a person has seen or talked to a medical doctor during the last year. For this variable, we classify as vulnerable people who have not seen or spoken to a doctor within the last year or who have seen or spoken to a doctor at least 52 times a year, that is, on average, once a week. Therefore, we consider as vulnerable people those who do not care about their health, as well as those who appear to have a medical problem. The second variable considered for this subindicator is the number of night stays in hospital during the last year, for which the selected threshold was one. We added these two binary variables to compute the Health care subindicator.

**Figure 3:**
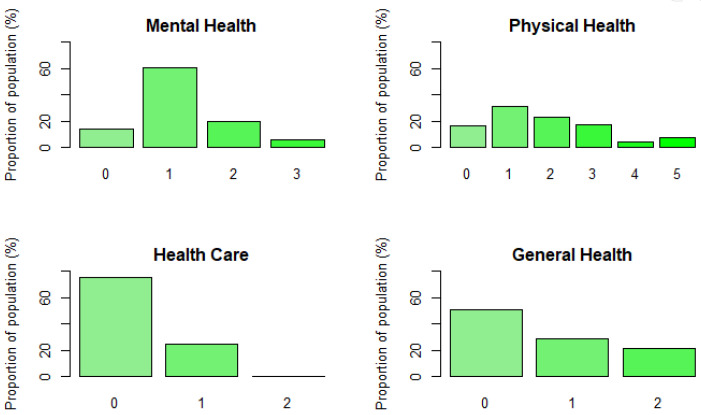
Health subindicators distribution.

The distribution of each subindicator is shown in Figure 3.

**Figure 4:**
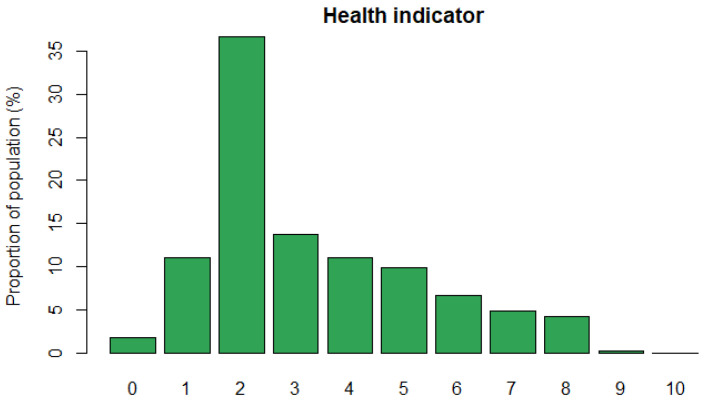
Health indicator distribution.

The health indicator is created by adding the previous health subindicators and rescaling to 0–10. In Figure 4, we depict the health indicator distribution. The mean value of this indicator is 3.28, the standard deviation is 0.01 and the median value is equal to 3. Considering the scale of this indicator, the previous measures allow us to understand that, in general, Europe has low levels of health vulnerability. However, 25% of the population is concentrated between levels 5 and 10 of vulnerability. A possible explanation is that health care expenditure among European countries is different. For instance, according to Eurostat [26], in 2017, the health care expenditure per capita in Switzerland was 8474 euros, while in Bulgaria, it was 550 euros.

### 2.2. Construction of the Profiles

The SHARE survey contains sociodemographic questions related to gender, age, marital status, and education. Here, we combine the information of these variables with the economic and health indicators to get the vulnerability profiles. For this purpose, we use a clustering algorithm which is able to cope with large datasets of weighted and mixed data. Due to the sampling design, the SHARE dataset includes a weighting variable, so that each individual represents a group of different size of the target population.

A very popular clustering algorithm is k-Means, which is fast and easy to interpret, but does not work with categorical data [27]. One possibility could be to use k-Medoids or Partitioning Around Medoids (PAM) with Gower’s distance. However, this algorithm is not efficient with a large datasets [28]. To circumvent the dimensionality problem, Clustering Large Applications (CLARA) can be used. This method uses a small sample of the dataset and applies PAM to generate the medoids and compute the final clusters. Since the first sample may not be representative, the method takes several samples, repeats the clustering process several times and the final result is selected considering the minimal average dissimilarity.

In this work, we prefer to use the k-Prototypes clustering algorithm by Huang [29], because it presents several advantages compared to the CLARA: (1) It works with the entire dataset instead of a sample, (2) It optimises the cost function using all the dataset, (3) CLARA takes a sample of the whole dataset, but the larger the dataset, the larger the sample size. Hence, if the sample is large, thousands of objects (as it is our case), the efficiency of CLARA will be affected [29].

As with any partitioning method, the number of clusters must be provided. Due to the large size of the dataset, we decide to use the “elbow” method, instead of the average silhouette width or other time-consuming criteria. This method consists of running the algorithm with a varying k and calculating the cost function for each run. Next, cost function values are plotted in a line-graph and at the point where there is a turning point, or “elbow”, the improvement in the cost function levels off. That is to say, the improvement by adding an additional grouping in the data is marginal, and the added complexity of understanding the profiles outweighs any benefits given.

On the k-prototypes algorithm.

Let $X_{1},X_{2},\;\ldots ,\;X_{p}$ be *p* numerical variables available in the dataset and $X_{p+1},X_{p+2},\;\ldots ,\;X_{m}$ be *m*-*p* categorical variables. Then, the dissimilarity between two individuals $x_{i},x_{j}\;\in \;{\mathbb{R}}^{p}$ is measured by a combination between the Euclidean and the Hamming distance:

(1) $$d\left(x_{i},x_{j}\right)=\overset{p}{\underset{h=1}{\sum }}{\left(x_{ih}-x_{jh}\right)}^{2}+\lambda \overset{m}{\underset{h=p+1}{\sum }}\delta \left(x_{ih},x_{jh}\right)$$

where δ(.,.) is the Hamming distance and λ≥0 is a parameter that measures the influence of numerical and categorical variables. When λ=0 the algorithm only considers the numerical ones.

This algorithm was implemented by Szepannek [30] in the *clustMixType* R-package. This package contains the function kproto that performs the following procedure:

1. Initialisation with random cluster prototypes,
2. For each observation do:
  (a) Assign observation to its closest prototype according to distance *d* (.,.)
  (b) Update cluster prototypes by cluster-specific means/modes for all variables
3. As long as any observations have swapped their cluster assignment in 2 or the maximum number of iterations has not been reached: repeat from 2 ([30], p. 201).

In this work, we introduce a particularity to the k-prototypes algorithm so that it can cope with weighted datasets. Thus, we modified function kproto accordingly with the help of the R-package (R Foundation for Statistical Computing, Vienna, Austria) survey by Lumley [21], which contains standard statistical techniques for weighted data. More details are given in the Appendix A.

### 2.3. Simulation of a Pandemic Shock

With the COVID-19 pandemic in 2020, we observed different shocks to health and economic systems. In developed countries, this crisis was different from previous crises, such as financial crisis of 2008, because it began with a health issue, that is, the economic crisis did not go directly to market failures, but was a consequence of having a quarantine in which economic sectors such as manufacturing, tourism and retail were affected. Therefore, to simulate a situation similar to COVID-19 pandemic, we design a shock that starts affecting a health variable and define which individuals are affected using proxy variables. Note that the surveys does not contains the information about which individuals are infected with COVID-19 to shock the features of those individuals directly.

National entities and organisations analysed what kind of population have more probability of being affected by this virus. For example, CDC [31] shows two groups of people at increased risk for severe illness: older adults and people with underlying medical conditions. According to WHO [32], that second group is formed by people affected by noncommunicable disease (NCDs) like cancer, diabetes, chronic respiratory disease, and cardiovascular disease.

During the pandemic, one of the recommendations for vulnerable population was isolation; some governments recommended implementing the telework to protect health and employment, but there are some jobs that cannot implemented in these ways, leading to risk of unemployment. On the other hand, the lockdown policies affected the economy, and many small and medium-sized enterprises (SMEs) have gone bankrupt, leading to an increase in the unemployment rate in Europe. Jones et al. [33] shows that in the United Kingdom 19% of workers were furloughed, in Germany this proportion was 23%, 29% in Italy and 14% in France.

Some publications mentioned the effects of this pandemic on mental health. For instance, Pieh et al. [34] studied the effects of Coronavirus on mental health in Austria. They found increases in depression, insomnia, anxiety and a decreases of quality life. Javed et al. ([35], p. 2) mentioned that “physical distancing due to the COVID-19 outbreak can have drastic negative effects on the mental health of the elderly and disables individuals”, these effects are stress, anxiety and depression. Schoeder [36] used the SHARELIFE survey to estimate the effect of involuntary job loss on health. He classified the job loss in two types, the first one as exogenous to the individual, like a plant closures, and the second one due to lay-offs. In his work, he found that the probability to be depressed increases when a man loses his job due to an exogenous cause. On the other hand, women had poorer general health; they reported more chronic conditions, and the probability to be obese or overweight increases.

**Figure 5:**
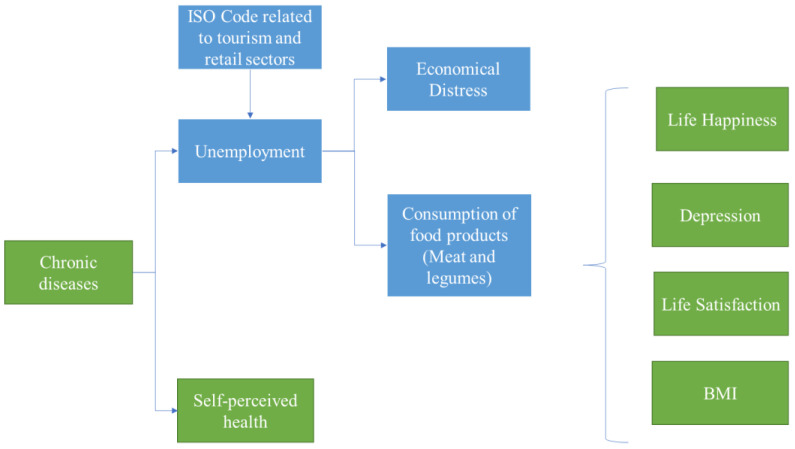
Shock design. Variables and sequence considered to simulate a pandemic shock.

The previous literature allows us to understand possible ways in which to design the shock. In this case, the shock is sequential, but all the changes of the variables occur at the same time. The shock design is shown in Figure 5 and explain it below.

The first step is to consider people who have chronic diseases. This variable produces an effect on the unemployed and self-perceived health subindicators in the following way: we consider that those people who had chronic diseases lost their job. Also those people with chronic diseases now they would perceive that their health is worse.

The coronavirus has not only affected the jobs of people with chronic diseases, but also jobs in certain economic sectors such as tourism and retail. Therefore, we suppose that people who worked in these sectors lose their jobs, due to this shock. We select the individuals affected by said shock, by means of the International Standard Classification of Occupations (ISCO 88) code related to tourism and retail sector.

The unemployed do not have the resources to be able to make ends meet and to consume the same quantity of foods like meat and legumes. For this reason, after the shock, those people are more vulnerable. Thus, individuals who become unemployed due to the coronavirus and before the shock were able to make ends meet, but now are struggling. For this reason, they are also considered vulnerable, which means that the subindicator Financial distress takes the value 1. Likewise, people who become unemployed due to the Coronavirus and before the shock had a value 0 in the weekly consumption subindicator, now after the shock this last value changed to 1.

Finally, the pandemic and those previous shocks could affect happiness and life satisfaction and increase depression and obesity or overweight, as we mentioned before. For this case, we consider two simultaneous shocks. The first, regarding the unemployed, with financial distress and vulnerable in terms of weekly consumption, will have a value 1 in the life happiness, depression, and life satisfaction variables. On the other hand, individuals with chronic diseases and with a worse self-perceived health, also will have a value 1 in those three variables.

The approach that we use to define the shock includes the idea that a pandemic crisis is different from an economic crisis, because, in this case, the shock starts with a health variable instead of an economic variable.

## 3. Results

In this section, we present the results of applying the construction of the indicators, the vulnerability profiles and the pandemic shock to the SHARE dataset. We start by visualising the indicators according to different groups established by sociodemographic information. Next, we visualise the vulnerability profiles across Europe and study the effect of the pandemic shock on them.

### 3.1. Visualising the Vulnerability Indicators

**Figure 6:**
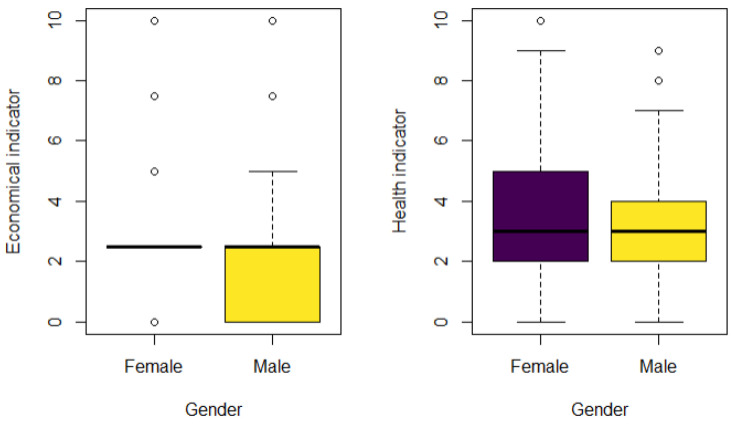
Boxplot economic and health indicators by gender.

In Figure 6, we depict the boxplots of these indicators by gender. The distribution of the economic indicator between male and female are different. For females, we can see that 50% of the observations are concentrated around 2.5, whereby the quartiles (Q1, Q2, Q3) are equal. While, the distribution of the economic indicator is negatively skewed, Q1 takes value 0 and Q3 is equal to 2, then 50% of the male population is located between 0 and 2. We infer that for both genders there are not many people with high economic vulnerability, nevertheless females are more economically vulnerable than males. These gender differences occur because of the gender employment and pay gap. The European Commission [37] estimated for 2017 that the gender employment gap was equal to 11%, and the gender pay gap was equal to 16%, for example, in Spain this gap is equal to 44.9% for females over 64 years old. For the health indicator, we do not observe differences in the median between males and females. Both median values are around 3, with a positively skewed distribution for females and a symmetric distribution for males. Also, there are possible outliers, which correspond to the most vulnerable cases. Van Oyen et al. [38] explains that this can happen because women have long life expectancy, which makes them reach ages where more diseases and mobility limitations appear.

**Figure 7:**
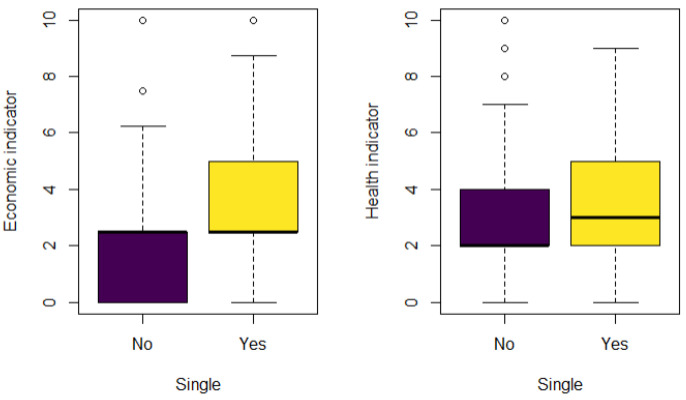
Boxplot economic and health indicators by marital status.

Next, we compare the distribution of our indicators by marital status. In Figure 7, we observe in the economic indicator that single people have a greater vulnerability level with a positively skewed distribution; in this case the median is around 2.5. People with a partner have a lower level of vulnerability and the distribution is negatively skewed. These differences occur because people with a partner may have more resources available for the household due to their jobs or pensions. Also, in the plot, we observe that people with a partner have better health than singles. The distribution for the people with a partner is positively skewed with a median of 2, while the distribution for single people is symmetric. People who live alone have more health risks due to physical condition: limited mobility, obesity and heavy smokers, according to Holt-Lunstad et al. [39], and also due to mental conditions like depression, stress and anxiety.

**Figure 8:**
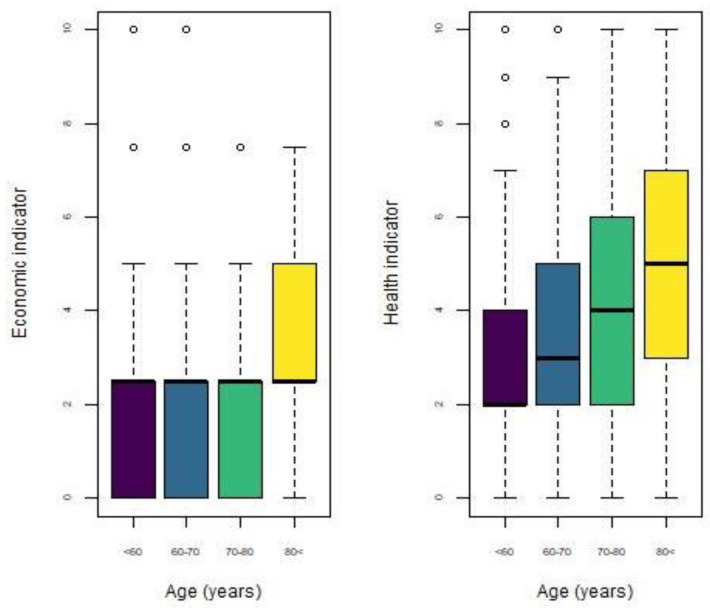
Boxplot of economic and health indicators by age group.

In Figure 8, both indicators are plotted by age group. Regarding the economic indicator, we observe that although all groups have the same median value, the distribution for people over 80 years old is positively skewed, and this group presents the greatest vulnerability levels. On the other hand, for the health indicator, it is observed that vulnerability level increases with age. The median value for people under 60 years old is 2, while that for people over 80 years old is 5. However, in the first two age groups, there are some outliers, indicating a high vulnerability level. Likewise, the shape of the distribution changes among groups, for example, the distribution for the youngest group is positively skewed, meanwhile, the distribution of the oldest group is symmetric. Above, we mentioned that life expectation has a negative correlation with health because the Healthy Life Years (HLY) are less, that is, people tend to have more limitations and chronic diseases as life goes on.

**Figure 9:**
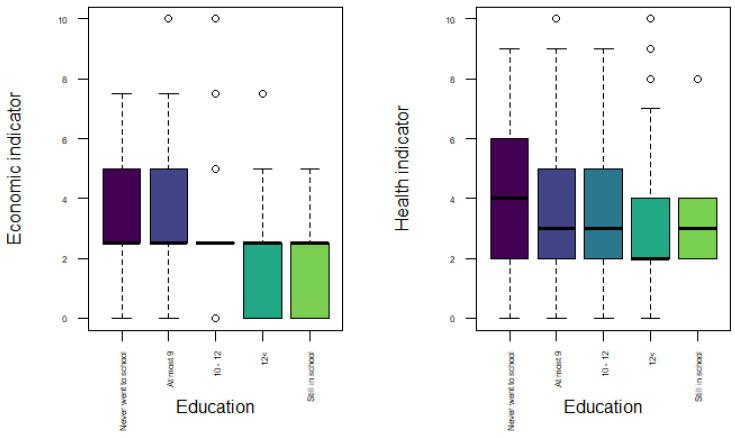
Boxplot of economic and health indicators by years of education.

In Figure 9, we compare both indicators according to years of education. The groups for years of education were selected using the quartiles and adding two groups for the people who never went to school and for those who were still studying. For the economic vulnerability indicator, we observe differences in terms of the distribution shape, but not in the median. In general, the vulnerability levels tend to decrease with the years of education. The distribution for the two first groups (people who never went to school and people that studied at most 9 years) are very similar, both groups reaching higher values of vulnerability. For the group who studied between 10 and 12 year, the values of vulnerability are concentrated around the median. The distribution is negatively skewed for those that studied more than 12 years, the 50% of the population older than 50 years old had economic vulnerability levels between 0 and 2.5. Also, people who said to be still studying have similar values of vulnerability than those with more years of education. The previous behaviour happens because the probability of being unemployed is reduced with more education [40], in addition there is a positive relation between the inequality years of education and inequality in income [41]. Regarding the health indicator, we observed more differences among groups. People who never went to school tend to have greater values of health vulnerability. When the years of education increase, the levels of vulnerability decrease until those people who studied more than 12 years. With these results, we can conclude that there are not many differences between people who studied an undergraduate degree or postgraduate degree in terms of health vulnerability.

The impact between education and health have been explained in two ways. The first is related to the economic indicator, in which we observed that more years of education implies more income; thus the more income, the more money that can be spent in health care. The second way is through mental and physical health. Zimmerman and Woolf [42] noted that people with more education develop more skills like reading, math and science that are related with better health, and also, people with higher education tend to do more exercise and healthy activities.

**Figure 10:**
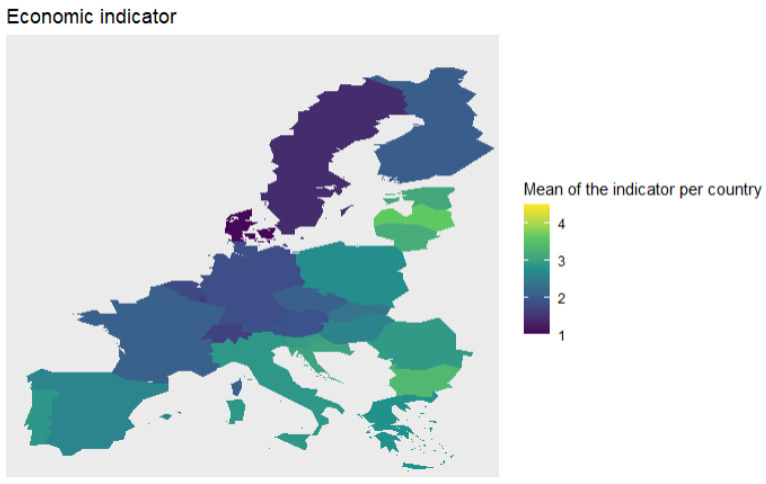
Average value of economic indicator per country.

**Figure 11:**
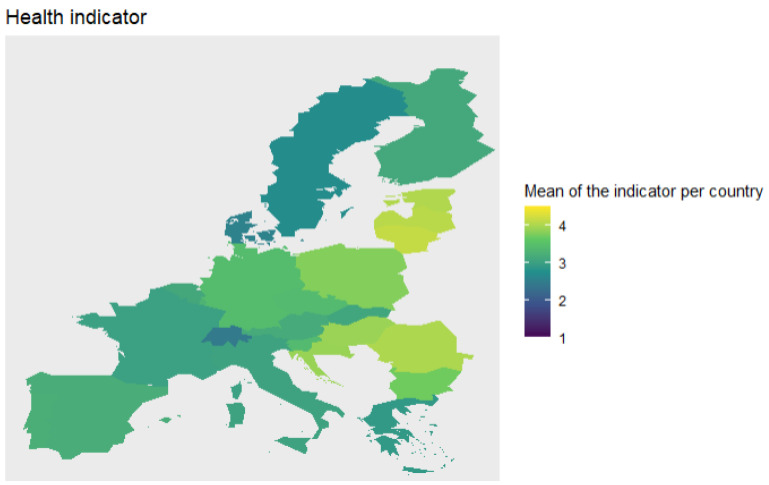
Average value of health indicator per country.

Finally, we compute the average value of each indicator per country and plot these values in a map to analyse both indicators across Europe (see Figure 10 and Figure 11).

When we plot the average value for the economic indicator per country, we can clearly see groups of countries. We observe that the Nordic countries have low levels of economic vulnerability, followed by the Central European region with levels around 2. Southern and Eastern Europe have similar levels of vulnerability, around 2.5. Finally, the Baltic region has the highest levels of vulnerability.

Likewise, we analyse the health indicator across Europe. Like the economic indicator, Nordic countries and Switzerland have the lowest levels of vulnerability. The next in the ranking are countries like France, Greece, Italy, and Portugal. On the other hand, the regions with highest level of vulnerability are Baltic and Balkan regions.

**Figure 12:**
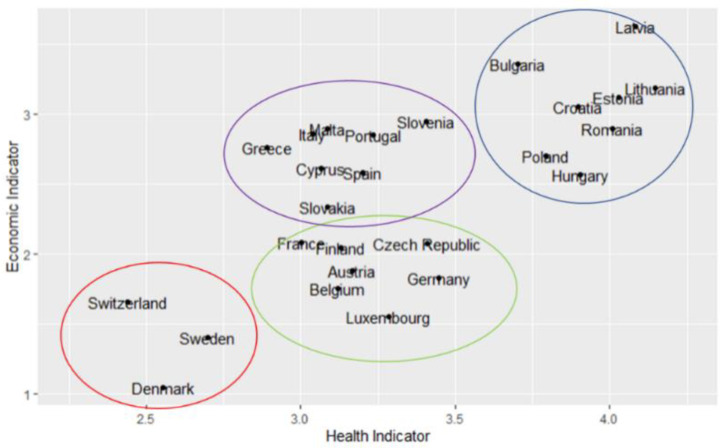
Economic vs. health indicator.

Finally, we want to understand the relationship between the two indicators in each country. For that purpose, we plot their mean values by country in Figure 12, where four groups of countries can be observed. In the lower left corner are located the Nordic countries, those with the lowest vulnerability level, according to the indicators created from the SHARE database. After that, in the centre of the graph are located two groups, the first one with Southern European countries like Portugal, Greece, Cyprus, Malta, Italy, Spain, and two Baltic countries like Slovenia and Slovakia, those countries have an average value between 3 and 3.5 in the health indicator and values around 2.5 in the economic indicator. The second group located in the centre is compounded by France and Finland with a very similar score in both indicators, near to 2 in the economic indicator and 3.25 in the health indicator. In the third group we found countries like Austria, Belgium, Germany, Luxembourg, and Czech Republic, which are characterised by having low levels of economic vulnerability, but a score greater than 3.5 in the health indicator. Finally, there is a group located in the top right corner, which basically consists of Baltic and Balkans states, whose countries have higher scores than the others in both indicators.

With the previous groups, we analyse the causes of these differences in the economic and health indicators. In Table 1, we report the mean and median values of the subindicators and indicators according to the groups described above. We can see that Southern European and Eastern regions have similar vulnerability levels of unemployment, although the Eastern region is more vulnerable due to the monetary poverty and financial distress. Northern European, Scandinavian regions, and Switzerland have the lowest levels of monetary poverty according with the SHARE data, but the unemployment reported in the Northern European countries is higher. For the health indicator, Eastern European countries have a great difference compared to the other regions in all the subindicators; however, these differences are more noticeable in the physical and mental health subindicators. The Northern European region has higher physical health vulnerability than Southern European, Scandinavian regions and Switzerland. On the other hand, the mean values of the health care subindicator of Scandinavian region and Switzerland are higher than those of Southern and Northern European countries, which means that the population of these regions have spoken to or seen a medical doctor more times or have stayed more nights at the hospital. Similar results to those previously described are observed using the median value. It is shown that at least 50% of the Southern, Northern and Eastern European population are vulnerable in terms of financial distress. Also, the median value of the health indicator is the highest for the Eastern European region, while the median value of mental health for the Southern, Northern and Eastern European regions is higher than corresponding one for Scandinavian countries and Switzerland.

### 3.2. Visualising the Vulnerability Profiles

**Figure 13:**
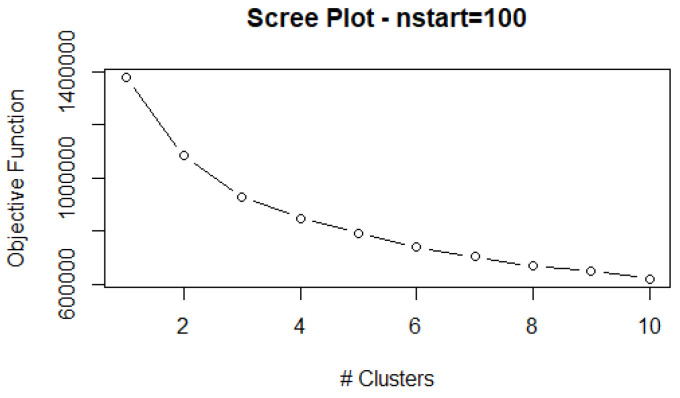
Scree plot.

Here, we cluster the individuals in groups with similar characteristics. To do so, we combine the economic and health vulnerability indicators with the sociodemographic information via a modified version of the k-Prototypes clustering algorithm. In order to select the number of clusters we run the algorithm for k = 2, 3, …, 10. In Figure 13 we show the cost function, where an “elbow” can be observed around k = 3 or k = 4. Finally, after investigating having both 3 or 4 profiles, we selected k = 4, since k = 3 led to too broad results.

**Figure 14:**
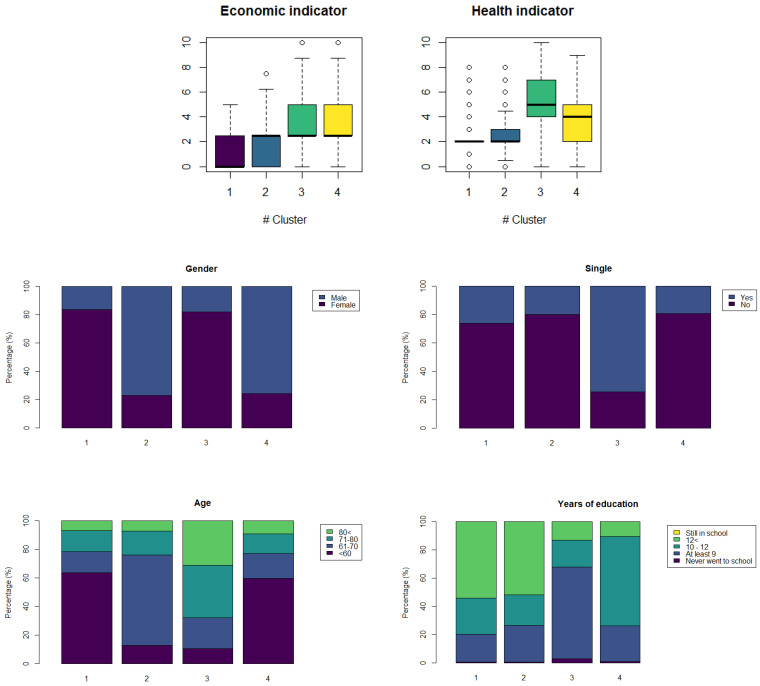
Distribution of each variable per cluster.

To define the profiles in terms of vulnerability, we analyse the distribution of each variable in each cluster. The distributions are shown in Figure 14.

Cluster 1 has low levels of economic vulnerability, with a maximum value close to 5. The health indicator has many instances with a vulnerability level of 2, but with some outliers with both very low and high levels of vulnerability. This cluster is characterised by females living with a partner, under 60 years old and with more than 12 years of education.

For cluster 2, the boxplot of the economic indicator shows that about 50% of the population has a vulnerability level between 0 and 2, but that there are people with higher level of economic vulnerability than in cluster 1. The distribution of the health indicator shows that at least half of the population has vulnerability levels between 2 and 3, with some outliers that reach higher levels, like 8. The profile of this cluster are males living with a partner, between 60 and 70 years old and more than 12 years of education.

The economic indicator in the cluster 3 has a positively skewed distribution with a median around 3, but 50% of the population is distributed between 3 and 5 level, also the values of this indicator reached values of 9 and it has some outliers with value 10 in the scale. For the health indicator in the same cluster, we find a median value of 5 and a mean of 5.21, both values are the highest among the clusters. In addition, this cluster is characterised by single females over 70 years old with at most 9 years of education.

Finally, in cluster 4, we observe a similar distribution in the economic indicator to that of cluster 3. The health indicator has a negatively skewed distribution with a median of 4 and a mean of 3.7. Likewise, 50% of the observations have a vulnerability level between 2 and 5. The profile of this cluster are males living with a partner, under 60 years old and with 10–12 years of education.

In conclusion, we can say that the most vulnerable profile is that found in cluster 3, since it presents both the highest levels of the health and economic indicators. The least vulnerable profile is that found in cluster 1. Clusters 4 and 2 define those profiles with a medium level of vulnerability. In summary, we can establish the following profiles sorted from the least to the most vulnerable: Profile 1 (Cluster 1), Profile 2 (Cluster 2), Profile 3 (Cluster 4), and Profile 4 (Cluster 3).

**Figure 15:**
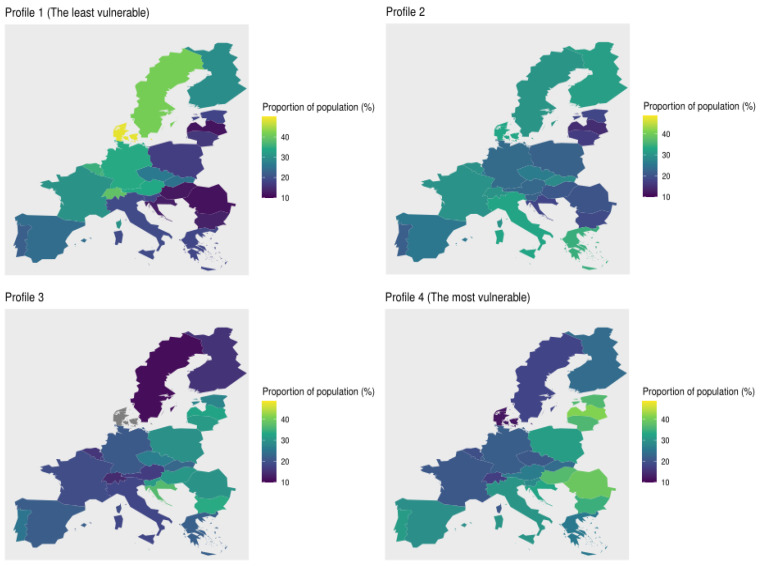
Proportion of population per country in each profile.

Next, we compute the proportion of the population per profile to analyse which countries have the most vulnerable people. We plot these maps in Figure 15. The countries with more population in the least vulnerable profile (Profile 1) are Denmark (48.4%), Sweden (41.2%), Switzerland (39.0%), Luxembourg (35.8%), Belgium (35.50%), and Germany (34.22%). Meanwhile, Baltic and Balkans states have less population in this profile, for instance, Romania (11.41%), Latvia (11.72%), Hungary (12.16%), and Croatia (12.93%). Also, in Profile 2 there is a high concentration of Scandinavian population, around 30%, but also countries as Greece (34.2%), Italy (32.4%), France (29.79%), Switzerland (29.7%), and Belgium (29.4%). In this profile the Baltic and Balkans states have a low concentration of population. This trend changes in Profile 3, where Eastern European countries have around 33% of their population, followed by countries like Poland (29.2%), Malta (28.1%), Estonia (27.5%), Czech Republic (26.5%), and Portugal (24.8%). On the other hand, Denmark, Sweden, and Switzerland have 6.8%, 10.8% and 14.3% of population within this cluster, respectively. Finally, concerning Profile 4, countries with more vulnerable population are Latvia (41.1%), Romania (20.1%), Hungary (36.68%), Estonia (36.3%), and Lithuania (36.0%), followed by Eastern European countries, like Poland (31.4%), and Southern European countries, like Portugal (31.0%), Italy (30.1%) and Spain (28.9%). In these profiles the top 5 of countries with less vulnerable population are Denmark (11.9%), Switzerland (16.8%), Sweden (17.7%), Belgium (19.3%), and France (20.7%).

Looking at the maps presented in Figure 15, we clearly observe that Eastern European countries are the most vulnerable, followed by Southern European countries. In third place are Central European countries, and finally, Scandinavian countries and Switzerland. Note that these findings are based on the geographical distribution of profiles 4 and 1, which are obtained from the available information in SHARE wave 7. Notice that some European countries (like Norwegian, Ireland, United Kingdom) were not included in the survey, thus, the inclusion of such countries might modify the previous findings.

### 3.3. Visualising the Pandemic Shock

**Figure 16:**
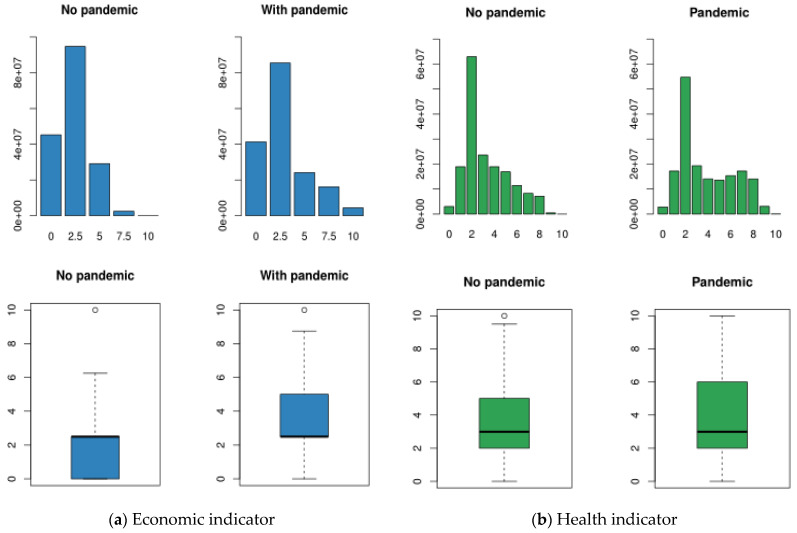
Bar-plot and boxplot of economic and health indicators before and after the pandemic.

Next, we apply the shock designed in Section 2.3 and again compute the economic and health indicators to analyse the differences. The distribution of the economic indicator moves to the right, as it has more observations with values of 7.5 and 10. This means that there are more vulnerable people. Also, we observed that 50% of the population now is located between 2.5 and 5, the median does not change, and the mean is 2.91 instead of 2.33. The Q1 value is equal to the Q3 value before the shock, this means that the pandemic had an economic impact. See panel (a) of Figure 16.

The health indicator also changes; the distribution moves to the right, but it still has a positively skewed distribution. As in the economic indicator, the median value does not change, but the mean is 3.86 instead of 3.2. Nevertheless, the Q3 is equal to 6, while before the shock this value was equal to 5 and after the shock a vulnerability level of 10 is not considered as an outlier. Finally, with a pandemic 50% of the European population older than 50 years old presents vulnerability levels between 2 and 6. See panel (b) of Figure 16.

**Figure 17:**
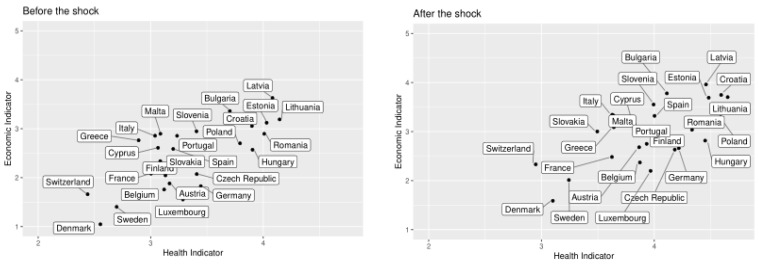
Relationship between economic and health indicator per country before and after the shock.

Next, we analyse how the countries were affected by the shock. In particular, we study how the groups sketched in Figure 12 are modified by the pandemic shock. These changes are illustrated in Figure 17.

Before the shock Denmark, Sweden, and Switzerland’s economic levels were around 1.5 and health levels around 2.5, while after the shock, they reach vulnerability levels around 2 and 3.25 in the economic and health indicators, respectively. This represents the highest growth rate in vulnerability levels after the shock. This is explained by the fact that before the pandemic these countries did not have a large population within the vulnerability groups, however, the after the pandemic shock those with the previously explained characteristics, such as chronic conditions, working sector and so on (see Figure 5) became vulnerable. Nevertheless, these three countries are still far apart on the scale of vulnerability from the other groups of countries.

The group comprising France, Finland, Luxembourg, Austria, Belgium, Germany, and Czech Republic experiment a high growth in their vulnerability levels. This means that these countries are more sensitive to health shocks that affect the mental health of their population. For example, before the shock, in Germany, the mean value of the mental health subindicator was 1.12, while after the shock, this value was 1.77. Meanwhile, the health indicator value preshock was 3.44, and after the shock it was 4.18. It is easy to see that the difference in the value of the health indicator is almost entirely explained by the variation in the mental health subindicator.

A third group was made up of Slovenia and Southern European countries like Spain, Portugal, Italy, Greece, and Malta. These countries still have greater economic vulnerability than Scandinavian and Central European countries, but the differences seem to remain in absolute terms. Additionally, countries like Spain and Greece present a great growth in their levels of health vulnerability, around 25.3% and 23.9%, respectively. The economy of these countries depends on sectors highly affected by the pandemic like tourism; this could increase the effect of the pandemic on their health vulnerability.

Finally, Eastern European countries move to the upper right corner but with a smaller magnitude. For instance, countries such as Romania, Hungary, and Bulgaria are more economically vulnerable after the shock, but the change is only of 3.6%, 8.2% and 9.89%, respectively. Likewise, these countries have the highest levels of health vulnerability, but also their growth rates are the lowest among European countries; for example, the growth rate of Romania, Latvia, and Bulgaria is 7.8%, 8.9% and 10.1%, respectively. Although Eastern European countries have less variation in their economic and health vulnerability, these countries are still the most vulnerable. Actually, this is not a good result because it means that in the preshock world these countries were very vulnerable, there is a high inequality in economic and health terms between Eastern European countries and the rest of Europe. 

**Figure 18:**
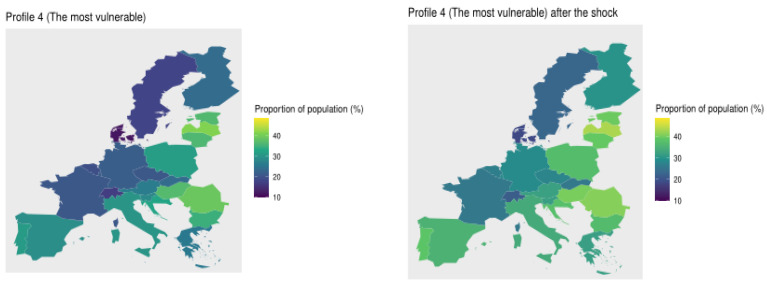
Geographical distribution of the most vulnerable profile before and after the shock.

**Figure 19:**
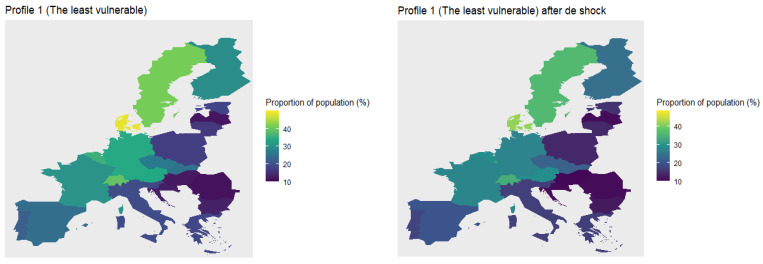
Geographical distribution of the least vulnerable profile before and after the shock.

With the new indicators, we recalculate the vulnerability profiles to analyse their variation after the shock. To compute the new profile composition, we use the predict function of the *clustMixType* R-package, and then we calculate the proportion of population that belongs to each profile and we plot it on a map. In Figure 18 and Figure 19 we illustrate the changes in the geographical distribution of the most and least vulnerable profiles, respectively.

After the shock, all countries have more population in the most vulnerable profile. Nevertheless, Scandinavian countries and Switzerland remain the least vulnerable populations. Southern European countries are more vulnerable after the shock. We observed a high change in the proportion of population in countries like Portugal (7.1%), Spain (6.2%), and Greece (6.5%). The proportion of the most vulnerable population in northern European countries also grew rapidly after the shock, for instance, Belgium’s growth rate was of 7.3%, Germany’s 7.17%, Luxemburg’s 6.9%, and Czech Republic’s 6.7%. Finally, Eastern European countries have more vulnerable population but the changes of in these countries were less abrupt. In countries like Romania the vulnerable proportion grew by 1.8%, in Latvia by 1.9%, and in Bulgaria by 2.2%.

The effect on the least vulnerable profile is similar. The proportion of population in these profiles decreases in each country. For instance, the proportion of population of Denmark and Sweden in this profile decreases around 7 and 5 percentage points, respectively, while the proportion of population of Romania and Latvia in this profile decreases around 0.5 and 0.9 percentage points, respectively. However, the effect of the pandemic is different among Eastern European countries; before the shock, the country with the lowest proportion of population in this profile was Romania (before the shock 11.4% and after the shock 10.8%), while afterwards it was Hungary (before the shock 12.1% and after the shock 10.4%).

The previous results allow us to understand that a health shock like a pandemic with economic and health consequences affect all European countries, but it affects more those countries with high levels of vulnerability in physical and mental health. Additionally, we observe two types of effects, i.e., countries that were previously more vulnerable do not experiment a high change in vulnerability, while countries with medium vulnerability profiles present a higher variation after the shock and their proportion of population in the most vulnerable profile tends to increase.

Future research and public health policies need to pay special attention to vulnerable populations including women, old people, the unemployed and those who have or had COVID-19 symptoms among others [15].

Due to the large proportion of the population affected by COVID-19, public health policies need to take health consequences into account [15]. For instance, the increase in loneliness related to social isolation during COVID-19 is linked to long-term health outcomes including all-cause mortality [43,44].

According to [45] this analysis of European countries’ vulnerability to the COVID-19 pandemic has identified the areas in which nations need to bolster their investments in order to alleviate the severity of the impact. Both domestic and EU funds should be allocated to these areas in order to ensure that their citizens are safe from the disease and, above all, protected from the related economic consequences.

## 4. Discussion

The elderly population in Europe constitutes a growing vulnerable group that is facing some economic and health challenges. Nowadays, the COVID pandemic is affecting the world health, economy, and daily life, being older people and those with NCDs the most affected. The objective of this work was to analyse the effects of a health shock on the older population’s well-being.

In the first part of this study, we used information from a survey applied to the European population aged 50 or older to design indicators that would allow us to measure the risk of being economically vulnerable and unhealthy. The first indicator was compounded by four subindicators: Monetary Poverty, Unemployment, Weekly Consumption, and Financial Distress, while the second one included Mental Health, Physical Health, Health Care, and General Health measures. Using these indicators, we concluded that the risk of vulnerability in Europe is low, because the mean value of the economic indicator is 2.33 and the median is 2.5, while the mean and median of the health indicator are 3.28 and 3, respectively—all values in a range from 0 to 10. However, the risk of vulnerability is not homogenous in the European territory, as it varies substantially among European regions. For instance, the Scandinavian countries and Switzerland have the lowest vulnerability levels in both indicators, while the Eastern European countries have the highest levels. The difference between regions is explained by the financial distress, the monetary poverty, and their vulnerability in mental and physical health.

Next, we combine the indicators with sociodemographic information in order to obtain the vulnerability profiles. This was done by using clustering techniques that allowed the use of quantitative variables, like the indicators, and categorical variables, such as gender, marital status, age groups, and education. More specifically, we used the k-prototypes clustering algorithm and modified it so that it can cope with weighted datasets. We found four profiles, where the most vulnerable profile is characterised by single women over 71 years old and with few years of education, more specifically with at most 9 years of education. Furthermore, Eastern European countries have the largest population within the most vulnerable profile, around a 38% of their population is older than 50 years old.

These results allowed us to achieve our objective, nevertheless, it was necessary to measure the effect of the pandemic. At the time this work was written, the effects of the COVID are still not clear, therefore, we designed a shock based on the available literature. This shock began affecting those individuals with chronic diseases. From this variable an effect was triggered onto variables such as individuals’ self-perception of health, life satisfaction, depression, happiness, and BMI. Additionally, the shock affected economic variables such as unemployment, which was most impactful in the tourism and retail sectors.

After the shock, we observed that a pandemic with the previous characteristics had effects in the health and economics of all European countries, which means their well-being is lower; however, it mostly affected countries where their population is more vulnerable in terms of physical and mental health. On the other hand, countries where the strongest economic sectors were affected, the negative effect on the health indicator was greater, for example, Spain and Greece.

Although the relative effect of the shock was lower in the Eastern European countries, the levels of economic and health vulnerability were high compared to the other European regions. This means that there is an inequality in Europe that should be reduced if European authorities want to reach the UNs 2030 Agenda for Sustainable Development.

The work presented herein is relevant, because it allowed us to characterise the most vulnerable populations, which is useful for public policies design and to better understand how these policies should be oriented differently in each region, according to the characteristics found in this study. For instance, it is important to design policies aimed towards mental and physical care, because these are variables that not only affect the Eastern European countries, but also, the Southern and Northern European countries. Likewise, this document suggests the study of public policies that help to reduce the financial distress in the Eastern European countries, since this was a variable in which there were notable differences with other European regions.

This work highlights the importance of indicators as instruments for measuring real phenomena, and the use of these for decision-making and the development of effective policies related to socioeconomic and health issues.

Finally, this work provides a methodology for the creation of profiles and the measurement of the effects of shocks through clustering techniques that allow the use of numerical and categorical variables, as well as calibrated cross-sectional weights.

Future studies should measure the real Coronavirus effects on the European population and the magnitude in which the vulnerability profiles were affected. After we designed our shock, SHARE designed a questionnaire related to COVID-19, in which they asked questions related to the variables used in our shock, for example, health and health behaviour, mental health, infections and health care, changes in work and economic situation. With this information, it will be possible not only to measure the general effect, but also the specific effects of the variables that compounded the subindicators and the indicators. In this way, public policies will be more effective in alleviating the most extreme situations of the vulnerable population.

## 5. Limitations

The statistical analysis presented in this paper is limited to the information available during the first COVID-19 wave, regarding health and wellbeing status in the EU. This information was collected in wave 7 SHARE dataset. As a result, this limitation constitutes recoded variables, surveyed individuals and countries that agreed to participate.

The analysis might be extended to other geographical areas with more diverse characteristics in order to confirm the conclusions drawn here, in cases where similar datasets to the SHARE database exist.

## Figures and Tables

**Table 1:** Mean (median) value of each indicator and subindicator per European region.

**Region**	**Unemployed Subindicator**	**Poverty Subindicator**	**W** **eekly Consumption Subindicator**	**Financial Distress Subindicator**	**Economic Indicator**
Southern European	0.04 (0)	0.22 (0)	0.01 (0)	0.83 (1)	2.73 (2.5)
Eastern European	0.04 (0)	0.29 (0)	0.00 (0)	0.91 (1)	3.07 (2.5)
Northern European	0.03 (0)	0.16 (0)	0.01 (0)	0.56 (1)	1.86 (2.5)
Scandinavian and Switzerland	0.01 (0)	0.16 (0)	0.01 (0)	0.37 (0)	1.37 (2.5)
**Region**	**General Health Subindicator**	**Mental Health Subindicator**	**Health Care Subindicator**	**Physical Health Subindicator**	**Health Indicator**
Southern European	0.65 (1)	1.26 (1)	0.25 (0)	1.68 (2)	3.13 (3)
Eastern European	0.85 (1)	1.57 (2)	0.33 (0)	2.06 (2)	3.95 (4)
Northern European	0.67 (1)	1.15 (1)	0.23 (0)	1.87 (2)	3.22 (3)
Scandinavian and Switzerland	0.46 (0)	0.89 (1)	0.26 (0)	1.49 (1)	2.56 (2)

## Data Availability

Original data can be downloaded from http://www.share-project.org/ (accessed on 1 April 2020).

## Appendix A Dataset and R Code

The k-prototypes method combines numerical and categorical variables using two types of measures, the Euclidean distance for numerical variables and Hamming distance for categorical variables. And this last term is controlled by λ. Then, these are the main steps in the function that we modified using the R-package “Survey”.

The first step was to adapt the definition of parameter λ in Szepannek [30] to the weighted context, that is, the calculus of the variance for the numeric and categorical variables used to compute λ. In the case of the numeric variable instead of computing the variance using the function var, we used the function svyvar that return the population variance, then as in the unweighted version we compute the mean of the variance vector. For the case of categorical variables, we had to compute the frequency table for each categorical variable using the function svytable but using a for loop that allows the program to loop through each of the variables. As with the numerical variables, we calculated the mean of the vector variance. The final step was to keep both results and divided them to obtain λ as in the unweighted case.

The first prototypes and the measure between them are computed in the same way as the original function. We changed the way in which the function computes the new prototypes, in the case of numerical variable we compute the population mean using the *svymean*. For the categorical case, we create a function that returns the levels with the highest frequency in each cluster. It is important to mention that in this case we have to use a subset of the initial survey design because we have now subsamples (clusters) that have not been defined previously, so the computation that we are doing only works for a part of the survey. The same idea is used for the final update of the prototypes.

Also, we use the survey package to compute the size of each cluster, the sum over all within clusters distance (withinss) and the sum over all clusters (tot.withinss).

The dataset and the R code used to obtain the results of this study are located on the next website: https://davidmerchan04.github.io/Impact-of-pandemic-on-European-well-being/ (accessed on 20 September 2020).

In this github project, you will find:

- SHARE_W7.zip: File with the Dataset used to the TFM

**Final code R:** R file with to code used to compute all the results of the TFM.
